# Forecasted datasets of electric vehicle consumption on the electricity grid of Spain

**DOI:** 10.1016/j.dib.2020.105823

**Published:** 2020-06-09

**Authors:** Dora Cama-Pinto, Juan Antonio Martínez-Lao, Andres Felipe Solano-Escorcia, Alejandro Cama-Pinto

**Affiliations:** a Department of Computer Architecture and Technology, University of Granada, 18071 Granada, Spain; b Department Engineering. University of Almeria, Ctra. Sacramento, s/n, 04120 La Cañada de San Urbano, Almería. Spain; c Department of Computer Science and Engineering Electronic, Universidad de la Costa, Calle 58 No. 55-66, Barranquilla, Colombia

**Keywords:** Electric vehicle, Grid to vehicle charging, Electric charging system, Spain, Electricity grid, Electricity consumption, Electricity availability

## Abstract

The information included in this study were calculated on the basis of data provided by the Spanish electricity grid, for thirteen years between 2007 and 2019. This data includes: the average consumption demand on the Spanish electricity grid at national level, and its availability. Subsequently, the report looks at the number of electric vehicles that could be supported in the years 2020–2023, depending on the consumption demand and availably of the electricity grid for those future years. The data presented in the article refers to the research study: ‘Electric vehicles in Spain: An overview of charging systems’[1] and ‘Analysis of charging stations for electric vehicles in Spain’ [2].

**Specifications Table**

**Table:** 

**Subject**	Renewable Energy, Sustainability and the Environment
	Energy Engineering and Power Technology
	Fuel Technology
**Specific subject area**	In Spain, the transport sector was responsible for 41.6% of total energy consumption in 2015 [3] and road transport is currently the second largest source of CO_2_ emissions in the European Union (EU) [4]. With the evolution of battery storage capacity, the efficiency and autonomy of electric vehicles have accelerated their introduction worldwide and in Spain. In this sense, it is important to know the availability of the Spanish Electricity grid to determine the introduction of future electric vehicles that could support simultaneous charging.
**Type of data**	Table
**How data were acquired**	In Spain, the demand for electricity grid consumption is available in a database. This database was used to forecast the average demand for electricity consumption and its availability, as well as a forecast regarding the numbers of electric vehicles that could be charged in future years, based on current capacity.
**Data format**	Direct URL to data:
	Mendeley Data, http://data.mendeley.com/datasets/658jkcht9g/1
**Parameters for data collection**	The primary data was extracted from the Spanish electricity grid database. The database includes detailed information on daily consumption in Megawatts at national level.
**Description of data collection**	The primary data in the database register for the last thirteen years (2007–2019) are organized by time, day, month and year. This information is used to forecast the demand and availability of electricity consumption, as well as to establish the number of electric vehicles that could be simultaneously recharged in the future.
**Data source location**	Country: Spain
**Data accessibility**	With the article
**Related research article**	Author's name: Cama-Pinto Dora, Cama-Pinto Alejandro, Martínez-Lao Juan Antonio, Francisco G. Montoya, Maria G. Montoya, Francisco Manzano-Agugliaro. Title: Electric vehicles in Spain: An overview of charging systems. Journal: Renewable and Sustainable Energy Reviews. https://doi.org/10.1016/j.rser.2016.11.23

## Value of the Data

• This dataset can be used to evaluate the effectiveness of policies implemented to promote the use of electric vehicles in Spain, potentially useful to other researchers.
• The data presented in this paper can save time for researchers who need to use this information as a starting point for forecasting the availability of renewable energy in Spain on an hourly basis over the twelve months of the years, 2020,2021, 2022, 2023.
• The data provided in this paper can be used to complement studies on the introduction and integration of different renewable energy sources with Spanish electricity [5,6].
• The benchmarks can be used to the analysis in the mass introduction of electric vehicles in Spain.
• The information provided in the article is useful for research into the forecast/trend of consumption behaviour in the Spanish electricity network.

## 1 Data

The electrical capacity in kWh of eighteen models of electric vehicles are shown in the Table 1. Each of these models ranges between 15.2 and 95 kWh, and is on sale in Spain with prices below 85 thousand euros, being the German car BMW i3s (42.2 kWh) a vehicle with average electric capacity according to the mentioned criterion.

**Table 1:** EV models for sale in Spain.

Model	Battery Capacity (kWh)
Citröen C-Zero	15,2
Mitsubishi i-MiEV	15,2
Peugeot ion	15,2
Smart EQ Fortwo	17,6
Hyundai Ioniq Electric	28
Citröen e-Méhari	30
Renault Kangoo Z.E.	33
Volkswagen e-Golf	35,8
Hyundai Kona 100	39,2
Kia e-NIRO 100	39,2
Nissan Leaf	40
Renault ZOE	41
BMW i3s	42,2
Nissan Leaf *E* + 3.Zero	62
Hyundai Kona 150	64
Kia e-NIRO 150	64
Tesla Model 3 LR	75
Audi e-tron	95

The Spanish electricity grid provides raw data of samples with frequencies of 10 min, openly accessible to the public, on the demand for electricity consumption at the national level [7]. Each of these values is averaged with all the values of the month for a specific time and year and this is what we observe in the data set stored on the Mendeley data website (https://data.mendeley.com) in the Excel spreadsheet file: Resume_Raw_Data.xlsx. Subsequently, the average of each hour of the day per month between 2007 and 2019 is described in Table 2.

**Table 2:** Average electrical demand per month during 2007–2019 in Spain in Megawatt.

Hour	**Jan.**	**Feb.**	**Mar.**	**Apr.**	**May.**	**Jun.**	**Jul.**	**Aug.**	**Sep.**	**Oct.**	**Nov.**	**Dec.**
00–01	28.766	28.807	27.079	25.583	25.113	26.467	28.265	27.069	25.919	24.567	26.148	27.492
01–02	26.320	26.440	25.006	23.724	23.427	24.743	26.354	25.208	24.338	23.031	24.197	25.106
02–03	24.677	24.894	23.626	22.467	22.293	23.526	24.932	23.859	23.263	22.055	22.956	23.515
03–04	23.826	24.137	22.949	21.850	21.757	22.936	24.198	23.116	22.746	21.605	22.371	22.682
04–05	23.470	23.841	22.698	21.573	21.538	22.669	23.828	22.719	22.501	21.422	22.163	22.341
05–06	23.733	24.166	23.015	21.746	21.721	22.784	23.878	22.748	22.656	21.644	22.511	22.604
06–07	25.420	26.007	24.687	23.259	22.959	23.732	24.970	23.746	24.067	23.167	24.241	24.129
07–08	28.702	29.556	27.212	25.589	24.632	25.349	26.352	24.889	26.477	26.078	27.326	27.090
08–09	31.571	32.081	29.513	27.343	26.834	27.620	28.508	26.494	27.847	27.905	29.392	29.550
09–10	33.060	33.653	31.236	29.006	28.502	29.505	30.841	28.679	29.380	28.853	30.833	31.235
10–11	34.360	34.723	32.318	30.139	29.712	31.000	32.663	30.412	30.663	29.813	31.876	32.555
11–12	34.672	34.852	32.550	30.529	30.292	31.747	33.537	31.483	31.462	30.280	32.103	32.798
12–13	34.366	34.544	32.390	30.637	30.632	32.331	34.348	32.312	32.086	30.597	32.043	32.469
13–14	34.169	34.303	32.167	30.496	30.657	32.563	34.768	32.883	32.295	30.577	31.920	32.336
14–15	33.129	33.114	31.023	29.478	29.679	31.730	34.139	32.485	31.409	29.573	30.894	31.459
15–16	32.387	32.337	30.201	28.610	28.846	30.996	33.445	31.711	30.661	28.773	30.186	30.695
16–17	32.104	31.992	29.726	28.215	28.527	30.823	33.235	31.362	30.486	28.471	29.941	30.522
17–18	32.485	32.005	29.562	27.981	28.415	30.835	33.163	31.204	30.473	28.480	30.607	31.342
18–19	34.538	32.912	29.753	27.734	28.171	30.552	32.782	30.941	30.217	28.667	32.997	33.736
19–20	36.068	35.590	31.785	27.834	28.050	30.219	32.346	30.570	30.093	29.904	33.889	34.399
20–21	36.307	36.361	33.787	29.006	28.447	30.057	31.909	30.502	31.464	31.778	33.988	34.524
21–22	35.895	35.919	33.554	31.346	29.878	30.308	31.938	31.736	32.311	31.196	33.199	34.079
22–23	33.985	33.828	31.571	29.753	29.300	30.348	32.001	30.939	29.874	28.660	30.999	32.287
23–24	31.625	31.377	29.237	27.461	26.992	28.343	30.056	28.899	27.551	26.358	28.576	29.970

To obtain the values of the average monthly electrical availability during 2007–2019 in megawatts, we the following four steps:

1) The average for a specific time in a month between 2007 and 2019 from the file Resume_Raw_Data.xlsx is located the highest consumption value.
2) This highest consumption value is subtracted with all the values of step1 (average of the month between 2007 and 2019 for a specific time in a given month) and we obtain the availability every 10 min (the sampling frequency of the Spanish electricity network)
3) The lowest value of electricity availability in each hour is averaged over each of the twelve months between 2007 and 2019 for the same time, the result of each value is shown in Table 3.

**Table 3:** Average electrical availability per month during 2007–2019 in Megawatt.

Hour	**Jan.**	**Feb.**	**Mar.**	**Apr.**	**May.**	**Jun.**	**Jul.**	**Aug.**	**Sep.**	**Oct.**	**Nov.**	**Dec.**
00–01	6.574	6.685	6.154	5.410	5.099	5.684	6.060	5.232	6.297	6.847	7.082	6.059
01–02	9.191	9.186	8.364	7.244	6.785	7.290	7.757	7.086	7.817	8.437	9.200	8.640
02–03	11.136	11.033	10.022	8.798	8.164	8.772	9.459	8.697	9.132	9.626	10.719	10.519
03–04	12.294	12.090	10.957	9.631	8.913	9.584	10.427	9.660	9.828	10.266	11.520	11.643
04–05	12.805	12.530	11.335	10.024	9.221	9.936	10.942	10.184	10.166	10.538	11.844	12.152
05–06	12.343	12.082	10.783	9.669	8.882	9.694	10.805	10.072	9.819	10.079	11.252	11.697
06–07	9.936	9.746	8.462	7.473	7.354	8.655	9.519	8.714	7.760	7.841	8.763	9.515
07–08	6.277	5.893	6.088	5.492	5.379	6.429	7.784	7.754	5.681	4.845	5.769	6.249
08–09	4.328	3.861	3.792	3.550	3.201	4.183	5.374	5.584	4.250	3.810	4.076	4.479
09–10	2.622	2.336	2.245	2.046	1.707	2.431	3.150	3.495	2.707	2.645	2.673	2.585
10–11	1.666	1.451	1.492	1.191	708	1.193	1.648	1.997	1.579	1.892	1.937	1.695
11–12	1.600	1.519	1.468	1.066	334	678	972	1.167	977	1.556	1.903	1.691
12–13	1.877	1.773	1.664	916	0	90	148	312	398	1.244	1.972	1.977
13–14	2.045	1.942	1.758	1.023	41	0	0	0	308	1.267	2.001	2.121
14–15	2.790	2.855	2.580	1.733	713	530	364	200	872	2.010	2.760	2.673
15–16	3.830	3.933	3.677	2.851	1.785	1.562	1.246	1.028	1.914	3.050	3.743	3.685
16–17	4.168	4.326	4.235	3.322	2.186	1.774	1.525	1.530	2.166	3.447	4.061	3.993
17–18	3.372	4.350	4.458	3.560	2.305	1.760	1.605	1.701	2.173	3.474	2.627	2.250
18–19	613	2.949	3.881	3.859	2.558	1.991	1.874	1.924	2.394	3.026	268	205
19–20	77	310	919	3.502	2.634	2.364	2.394	2.285	2.387	989	22	26
20–21	0	0	0	1.518	1.977	2.571	2.757	2.193	241	0	0	0
21–22	15	13	64	0	372	1.905	2.489	876	0	177	267	62
22–23	1.605	1.774	1.715	871	654	1.903	2.470	1.187	1.743	2.222	2.196	1.509
23–24	3.826	4.067	3.930	3.332	2.908	3.445	3.919	3.283	4.332	4.844	4.533	3.691

The Table 4 determines the number of new EVs that can be simultaneously recharged in 60-min. intervals with a connection power of 50 kW, to this purpose, the values in Table 3 are divided by 50 kW, because it has an approximate value reference the BMW i3s (42.2 kWh) that appears in Table 1, to determine the amount of EV that would support the Spanish electric grid.

**Table 4:** EV amount that can be recharged from 0 to 24 h.

**Hour**	**Jan.**	**Feb.**	**Mar.**	**Apr.**	**May.**	**Jun.**	**Jul.**	**Aug.**	**Sep.**	**Oct.**	**Nov.**	**Dec.**
00–01	131.480	133.696	123.089	108.210	101.990	113.671	121.199	104.648	125.937	136.937	141.632	121.177
01–02	183.819	183.715	167.272	144.870	135.703	145.793	155.140	141.711	156.346	168.742	183.991	172.800
02–03	222.712	220.664	200.432	175.963	163.273	175.437	189.190	173.937	182.645	192.521	214.372	210.390
03–04	245.871	241.808	219.147	192.628	178.263	191.680	208.546	193.193	196.563	205.320	230.403	232.859
04–05	256.090	250.603	226.706	200.472	184.416	198.718	218.848	203.689	203.311	210.755	236.873	243.038
05–06	246.856	241.646	215.654	193.386	177.638	193.879	216.091	201.435	196.385	201.585	225.032	233.934
06–07	198.722	194.920	169.241	149.463	147.076	173.102	190.390	174.276	155.195	156.812	175.263	190.297
07–08	125.536	117.852	121.757	109.835	107.573	128.587	155.687	155.081	113.622	96.902	115.390	124.982
08–09	86.554	77.211	75.830	70.998	64.019	83.669	107.482	111.684	85.002	76.205	81.511	89.585
09–10	52.432	46.729	44.900	40.922	34.138	48.616	63.009	69.909	54.132	52.895	53.470	51.697
10–11	33.329	29.030	29.830	23.814	14.159	23.853	32.956	39.931	31.580	37.840	38.744	33.901
11–12	31.998	30.387	29.351	21.324	6.683	13.550	19.431	23.336	19.550	31.119	38.060	33.813
12–13	37.537	35.464	33.288	18.323	0	1.794	2.951	6.242	7.969	24.877	39.431	39.531
13–14	40.904	38.848	35.153	20.463	816	0	0	0	6.152	25.340	40.027	42.411
14–15	55.806	57.102	51.598	34.663	14.263	10.608	7.272	3.997	17.440	40.196	55.191	53.467
15–16	76.602	78.657	73.544	57.011	35.692	31.241	24.913	20.566	38.271	61.004	74.858	73.696
16–17	83.350	86.523	84.709	66.445	43.724	35.482	30.504	30.604	43.313	68.933	81.227	79.867
17–18	67.434	86.993	89.163	71.207	46.104	35.204	32.097	34.020	43.453	69.479	52.536	45.008
18–19	12.260	58.971	77.613	77.171	51.150	39.823	37.471	38.484	47.888	60.517	5.361	4.101
19–20	1.532	6.190	18.371	70.045	52.670	47.280	47.880	45.691	47.739	19.780	431	514
20–21	0	0	0	30.351	39.548	51.412	55.143	43.860	4.827	0	0	0
21–22	309	265	1.270	0	7.443	38.098	49.772	17.528	0	3.539	5.335	1.235
22–23	32.102	35.473	34.298	17.414	13.078	38.060	49.407	23.731	34.866	44.430	43.914	30.173
23–24	76.513	81.331	78.607	66.639	58.156	68.895	78.387	65.658	86.632	96.883	90.650	73.811

From the file Resume_Raw_Data.xlsx(http://data.mendeley.com/datasets/658jkcht9g/2), we build the forecasts for Average electrical demand (Megawatt) per month during 2020, 2021, 2022, 2023 in Spain (Tables 5, 6, 7, 8), then, from the data in those tables we developed Tables 9, 10, 11, 12, which are the availability of electricity in megawatts for the years between 2020 and 2023. Finally, this information leads to the elaboration of EV amount that can be recharged simultaneously from 0 to 24 h during 2020,2021,2022,2023 in the Table 13,14,15,16 respectively.

**Table 5:** Forecast for Average electrical demand (Megawatt) per month during 2020 in Spain.

**Hour**	**Jan.**	**Feb.**	**Mar.**	**Apr.**	**May.**	**Jun.**	**Jul.**	**Aug.**	**Sep.**	**Oct.**	**Nov.**	**Dec.**
00–01	26.383	26.850	25.500	24.439	24.408	26.167	27.997	27.092	25.602	23.928	24.681	24.792
01–02	24.458	24.965	23.852	22.925	23.139	24.862	26.498	25.532	24.410	22.794	23.224	23.050
02–03	23.180	23.766	22.808	21.944	22.221	23.831	25.282	24.344	23.490	21.983	22.277	21.877
03–04	22.625	23.312	22.383	21.509	21.792	23.320	24.623	23.678	23.047	21.597	21.860	21.326
04–05	22.533	23.247	22.300	21.366	21.619	23.079	24.255	23.313	22.790	21.428	21.771	21.218
05–06	23.080	23.800	22.832	21.694	21.874	23.236	24.331	23.374	22.951	21.671	22.238	21.676
06–07	25.042	25.868	24.684	23.308	23.129	24.185	25.376	24.371	24.254	23.066	24.070	23.367
07–08	28.571	29.595	27.381	25.566	24.844	25.834	26.730	25.538	26.388	25.614	27.086	26.374
08–09	30.803	31.617	29.506	27.219	26.728	27.561	28.428	26.953	27.379	26.892	28.839	28.252
09–10	32.414	33.214	31.171	28.850	28.155	29.232	30.575	29.046	28.868	28.011	30.312	29.909
10–11	33.389	33.886	31.783	29.610	28.867	30.272	31.940	30.458	29.783	28.726	31.025	30.895
11–12	33.563	33.895	31.877	29.832	29.263	30.884	32.710	31.456	30.522	29.222	31.144	31.004
12–13	33.262	33.645	31.638	29.887	29.588	31.486	33.632	32.369	31.270	29.628	31.124	30.719
13–14	33.227	33.603	31.478	29.868	29.733	31.845	34.133	33.054	31.775	29.815	31.214	30.715
14–15	32.522	32.810	30.666	29.152	29.095	31.412	33.965	32.991	31.320	29.211	30.520	30.154
15–16	31.630	31.908	29.757	28.145	28.185	30.666	33.341	32.352	30.594	28.367	29.685	29.289
16–17	31.059	31.352	29.100	27.522	27.661	30.344	33.001	31.969	30.293	27.907	29.161	28.805
17–18	31.201	31.135	28.783	27.127	27.434	30.198	32.798	31.704	30.074	27.698	29.390	29.217
18–19	32.664	31.657	28.820	26.871	27.218	29.895	32.352	31.331	29.692	27.578	30.991	30.785
19–20	34.096	33.996	30.654	27.086	27.280	29.760	32.052	30.929	29.507	28.502	31.813	31.439
20–21	34.634	34.877	32.366	28.161	27.768	29.759	31.749	30.757	30.552	30.023	32.193	31.805
21–22	34.131	34.381	31.963	29.843	28.750	29.815	31.622	31.441	31.091	29.490	31.445	31.255
22–23	32.024	32.115	30.048	28.215	27.769	29.227	31.107	30.416	28.704	27.192	29.172	29.325
23–24	28.748	28.911	27.308	26.230	25.787	27.405	29.297	28.675	26.746	25.247	26.589	26.725

**Table 6:** Forecast for Average electrical demand (Megawatt) per month during 2021 in Spain.

**Hour**	**Jan.**	**Feb.**	**Mar.**	**Apr.**	**May.**	**Jun.**	**Jul.**	**Aug.**	**Sep.**	**Oct.**	**Nov.**	**Dec.**
00–01	26.042	26.570	25.275	24.276	24.307	26.125	27.959	27.095	25.556	23.836	24.471	24.406
01–02	24.192	24.755	23.688	22.811	23.098	24.879	26.518	25.578	24.420	22.761	23.085	22.756
02–03	22.967	23.605	22.691	21.869	22.211	23.874	25.332	24.414	23.522	21.973	22.180	21.643
03–04	22.453	23.194	22.302	21.460	21.797	23.375	24.684	23.759	23.090	21.596	21.787	21.133
04–05	22.399	23.162	22.243	21.337	21.631	23.138	24.316	23.398	22.831	21.429	21.715	21.057
05–06	22.986	23.748	22.806	21.687	21.896	23.300	24.396	23.463	22.993	21.675	22.199	21.543
06–07	24.988	25.848	24.683	23.315	23.153	24.250	25.434	24.460	24.281	23.052	24.045	23.258
07–08	28.552	29.600	27.405	25.563	24.875	25.903	26.784	25.631	26.376	25.548	27.051	26.271
08–09	30.693	31.551	29.506	27.201	26.713	27.552	28.417	27.018	27.312	26.747	28.760	28.066
09–10	32.322	33.151	31.162	28.827	28.106	29.193	30.537	29.099	28.795	27.891	30.237	29.719
10–11	33.250	33.767	31.706	29.535	28.746	30.169	31.836	30.465	29.657	28.570	30.903	30.658
11–12	33.405	33.758	31.780	29.732	29.115	30.760	32.591	31.452	30.388	29.071	31.007	30.748
12–13	33.104	33.517	31.531	29.779	29.439	31.366	33.530	32.377	31.153	29.490	30.992	30.469
13–14	33.092	33.503	31.379	29.778	29.601	31.743	34.042	33.079	31.700	29.706	31.113	30.484
14–15	32.436	32.767	30.615	29.106	29.011	31.367	33.940	33.064	31.307	29.160	30.466	29.968
15–16	31.522	31.846	29.694	28.079	28.090	30.619	33.326	32.443	30.584	28.308	29.614	29.089
16–17	30.910	31.261	29.011	27.423	27.538	30.276	32.967	32.056	30.265	27.827	29.050	28.560
17–18	31.018	31.011	28.671	27.005	27.293	30.107	32.746	31.776	30.018	27.586	29.216	28.914
18–19	32.397	31.478	28.686	26.748	27.082	29.802	32.290	31.387	29.617	27.422	30.704	30.364
19–20	33.815	33.768	30.493	26.979	27.170	29.694	32.010	30.980	29.424	28.302	31.516	31.016
20–21	34.396	34.665	32.163	28.040	27.671	29.716	31.726	30.794	30.421	29.772	31.937	31.417
21–22	33.879	34.161	31.736	29.629	28.588	29.744	31.576	31.399	30.917	29.246	31.194	30.851
22–23	31.743	31.871	29.830	27.995	27.550	29.067	30.979	30.342	28.537	26.982	28.911	28.902
23–24	28.337	28.559	27.033	26.054	25.615	27.271	29.189	28.643	26.631	25.088	26.306	26.261

**Table 7:** Forecast for Average electrical demand (Megawatt) per month during 2022 in Spain.

**Hour**	**Jan.**	**Feb.**	**Mar.**	**Apr.**	**May.**	**Jun.**	**Jul.**	**Aug.**	**Sep.**	**Oct.**	**Nov.**	**Dec.**
00–01	25.702	26.291	25.049	24.113	24.207	26.082	27.920	27.098	25.511	23.745	24.261	24.021
01–02	23.926	24.544	23.523	22.697	23.057	24.896	26.539	25.624	24.430	22.727	22.945	22.462
02–03	22.753	23.443	22.574	21.794	22.201	23.918	25.382	24.483	23.555	21.963	22.082	21.409
03–04	22.282	23.076	22.221	21.412	21.802	23.430	24.745	23.839	23.133	21.595	21.714	20.939
04–05	22.265	23.077	22.186	21.307	21.643	23.197	24.377	23.483	22.872	21.430	21.659	20.897
05–06	22.893	23.695	22.780	21.679	21.917	23.365	24.461	23.552	23.035	21.679	22.160	21.411
06–07	24.934	25.828	24.683	23.321	23.177	24.314	25.492	24.550	24.308	23.037	24.020	23.149
07–08	28.533	29.605	27.429	25.559	24.905	25.972	26.838	25.724	26.363	25.482	27.017	26.169
08–09	30.583	31.484	29.505	27.183	26.697	27.544	28.405	27.084	27.245	26.602	28.681	27.881
09–10	32.230	33.088	31.153	28.805	28.056	29.154	30.500	29.151	28.722	27.771	30.163	29.530
10–11	33.112	33.647	31.630	29.459	28.625	30.065	31.733	30.472	29.532	28.415	30.781	30.421
11–12	33.246	33.621	31.684	29.632	28.968	30.637	32.473	31.448	30.254	28.920	30.870	30.492
12–13	32.947	33.388	31.423	29.672	29.290	31.245	33.428	32.385	31.037	29.352	30.861	30.219
13–14	32.958	33.403	31.281	29.689	29.469	31.640	33.951	33.103	31.626	29.598	31.012	30.252
14–15	32.349	32.724	30.564	29.059	28.928	31.321	33.915	33.136	31.294	29.108	30.413	29.781
15–16	31.414	31.785	29.630	28.012	27.996	30.572	33.311	32.535	30.575	28.250	29.542	28.888
16–17	30.761	31.170	28.922	27.323	27.414	30.207	32.934	32.143	30.237	27.746	28.938	28.314
17–18	30.834	30.886	28.560	26.883	27.153	30.016	32.694	31.847	29.961	27.475	29.042	28.610
18–19	32.129	31.299	28.553	26.625	26.945	29.708	32.229	31.443	29.542	27.267	30.418	29.942
19–20	33.533	33.541	30.331	26.872	27.060	29.629	31.968	31.032	29.340	28.101	31.220	30.593
20–21	34.157	34.453	31.960	27.920	27.574	29.673	31.703	30.830	30.291	29.521	31.680	31.028
21–22	33.627	33.942	31.508	29.414	28.427	29.674	31.531	31.356	30.743	29.002	30.944	30.448
22–23	31.463	31.626	29.612	27.776	27.332	28.907	30.852	30.267	28.370	26.773	28.650	28.478
23–24	27.925	28.206	26.757	25.878	25.443	27.137	29.080	28.611	26.516	24.930	26.022	25.797

**Table 8:** Forecast for Average electrical demand (Megawatt) per month during 2023 in Spain.

**Hour**	**Jan.**	**Feb.**	**Mar.**	**Apr.**	**May.**	**Jun.**	**Jul.**	**Aug.**	**Sep.**	**Oct.**	**Nov.**	**Dec.**
00–01	25.361	26.011	24.824	23.949	24.106	26.039	27.882	27.102	25.466	23.654	24.051	23.635
01–02	23.660	24.333	23.358	22.583	23.016	24.913	26.559	25.671	24.440	22.693	22.806	22.169
02–03	22.539	23.282	22.457	21.720	22.191	23.961	25.432	24.552	23.587	21.953	21.985	21.175
03–04	22.110	22.958	22.140	21.363	21.807	23.485	24.805	23.920	23.176	21.594	21.641	20.745
04–05	22.131	22.993	22.130	21.278	21.654	23.255	24.438	23.568	22.913	21.431	21.603	20.736
05–06	22.800	23.643	22.754	21.672	21.939	23.429	24.526	23.642	23.077	21.683	22.121	21.278
06–07	24.880	25.809	24.682	23.328	23.201	24.379	25.550	24.639	24.334	23.023	23.996	23.040
07–08	28.515	29.611	27.453	25.556	24.935	26.041	26.891	25.817	26.350	25.416	26.983	26.067
08–09	30.474	31.418	29.504	27.165	26.682	27.535	28.394	27.149	27.178	26.458	28.602	27.695
09–10	32.137	33.025	31.144	28.782	28.006	29.115	30.462	29.204	28.649	27.650	30.088	29.341
10–11	32.973	33.528	31.553	29.384	28.504	29.961	31.630	30.478	29.406	28.260	30.660	30.184
11–12	33.088	33.485	31.588	29.533	28.821	30.514	32.355	31.445	30.119	28.769	30.733	30.235
12–13	32.789	33.260	31.316	29.565	29.141	31.124	33.326	32.394	30.920	29.213	30.730	29.969
13–14	32.823	33.303	31.182	29.599	29.337	31.538	33.861	33.127	31.552	29.489	30.911	30.021
14–15	32.263	32.681	30.513	29.012	28.844	31.276	33.890	33.209	31.281	29.056	30.360	29.595
15–16	31.306	31.724	29.567	27.946	27.901	30.525	33.297	32.627	30.565	28.192	29.471	28.687
16–17	30.612	31.078	28.833	27.224	27.290	30.139	32.900	32.229	30.210	27.665	28.827	28.069
17–18	30.651	30.762	28.449	26.761	27.013	29.925	32.642	31.919	29.904	27.363	28.868	28.307
18–19	31.861	31.119	28.420	26.502	26.809	29.614	32.167	31.498	29.467	27.111	30.131	29.521
19–20	33.251	33.313	30.170	26.765	26.950	29.563	31.926	31.083	29.257	27.901	30.923	30.170
20–21	33.918	34.241	31.757	27.799	27.477	29.631	31.680	30.867	30.160	29.270	31.424	30.640
21–22	33.375	33.722	31.281	29.200	28.266	29.603	31.486	31.314	30.568	28.759	30.693	30.045
22–23	31.183	31.381	29.395	27.556	27.113	28.747	30.724	30.193	28.203	26.563	28.389	28.055
23–24	27.514	27.854	26.482	25.703	25.271	27.002	28.972	28.579	26.401	24.771	25.738	25.334

**Table 9:** Electrical availability forecast (MW) from 0 to 24 h in 2020.

**Hour**	**Jan.**	**Feb.**	**Mar.**	**Apr.**	**May.**	**Jun.**	**Jul.**	**Aug.**	**Sep.**	**Oct.**	**Nov.**	**Dec.**
00–01	8.252	8.027	6.866	5.447	5.325	5.678	6.136	5.962	6.173	6.095	7.513	7.013
01–02	10.177	9.912	8.513	6.962	6.594	6.983	7.635	7.522	7.365	7.228	8.969	8.755
02–03	11.454	11.111	9.558	7.943	7.512	8.015	8.850	8.710	8.285	8.039	9.917	9.928
03–04	12.009	11.565	9.983	8.377	7.941	8.525	9.510	9.376	8.727	8.425	10.333	10.479
04–05	12.101	11.630	10.066	8.520	8.114	8.766	9.877	9.741	8.985	8.594	10.422	10.587
05–06	11.555	11.077	9.533	8.193	7.860	8.609	9.801	9.681	8.824	8.352	9.955	10.129
06–07	9.592	9.009	7.682	6.579	6.604	7.660	8.757	8.683	7.521	6.957	8.124	8.439
07–08	6.064	5.283	4.985	4.321	4.889	6.012	7.403	7.516	5.386	4.408	5.108	5.432
08–09	3.832	3.260	2.859	2.668	3.005	4.284	5.705	6.101	4.396	3.131	3.355	3.553
09–10	2.220	1.664	1.195	1.037	1.578	2.613	3.557	4.008	2.906	2.012	1.882	1.896
10–11	1.245	991	583	276	866	1.573	2.193	2.596	1.992	1.297	1.169	910
11–12	1.071	982	489	55	471	962	1.423	1.598	1.253	800	1.049	801
12–13	1.372	1.232	728	0	145	359	500	685	505	394	1.069	1.086
13–14	1.408	1.274	888	19	0	0	0	0	0	207	979	1.090
14–15	2.112	2.067	1.700	735	638	433	168	63	455	811	1.673	1.651
15–16	3.005	2.969	2.609	1.741	1.549	1.179	791	702	1.181	1.656	2.508	2.516
16–17	3.575	3.525	3.265	2.365	2.072	1.501	1.132	1.085	1.482	2.116	3.032	3.000
17–18	3.433	3.742	3.583	2.760	2.300	1.647	1.334	1.350	1.700	2.325	2.803	2.588
18–19	1.970	3.220	3.546	3.015	2.515	1.950	1.781	1.723	2.083	2.445	1.202	1.020
19–20	538	881	1.711	2.801	2.453	2.085	2.081	2.125	2.267	1.521	380	367
20–21	0	0	0	1.726	1.965	2.087	2.384	2.297	1.223	0	0	0
21–22	503	496	403	43	984	2.030	2.511	1.613	683	533	748	550
22–23	2.611	2.762	2.318	1.671	1.964	2.618	3.026	2.638	3.070	2.831	3.021	2.480
23–24	5.887	5.966	5.058	3.657	3.946	4.441	4.835	4.379	5.029	4.776	5.604	5.081

**Table 10:** Electrical availability forecast (MW) from 0 to 24 h in 2021.

**Hour**	**Jan.**	**Feb.**	**Mar.**	**Apr.**	**May.**	**Jun.**	**Jul.**	**Aug.**	**Sep.**	**Oct.**	**Nov.**	**Dec.**
00–01	8.353	8.095	6.888	5.503	5.294	5.618	6.083	5.983	6.144	5.936	7.466	7.010
01–02	10.204	9.911	8.475	6.968	6.503	6.864	7.524	7.501	7.281	7.011	8.852	8.661
02–03	11.429	11.061	9.472	7.910	7.390	7.869	8.710	8.665	8.178	7.799	9.757	9.773
03–04	11.942	11.471	9.861	8.319	7.805	8.368	9.358	9.320	8.610	8.176	10.150	10.284
04–05	11.996	11.503	9.919	8.443	7.970	8.605	9.726	9.680	8.869	8.343	10.222	10.359
05–06	11.409	10.917	9.356	8.093	7.706	8.442	9.646	9.616	8.707	8.097	9.738	9.873
06–07	9.407	8.817	7.480	6.465	6.448	7.493	8.608	8.618	7.419	6.720	7.892	8.159
07–08	5.843	5.065	4.758	4.217	4.727	5.840	7.258	7.447	5.325	4.224	4.885	5.146
08–09	3.702	3.115	2.657	2.578	2.889	4.190	5.625	6.060	4.388	3.025	3.177	3.350
09–10	2.074	1.514	1.001	952	1.496	2.550	3.504	3.980	2.905	1.881	1.700	1.697
10–11	1.145	898	457	245	855	1.574	2.205	2.614	2.043	1.201	1.034	759
11–12	991	907	382	47	486	982	1.451	1.626	1.312	701	929	669
12–13	1.291	1.148	632	0	162	377	512	701	547	282	944	948
13–14	1.303	1.162	784	1	0	0	0	0	0	65	824	933
14–15	1.960	1.898	1.548	674	590	376	102	15	393	612	1.470	1.449
15–16	2.874	2.819	2.469	1.701	1.511	1.123	716	635	1.116	1.463	2.323	2.328
16–17	3.485	3.404	3.152	2.357	2.064	1.467	1.075	1.023	1.435	1.945	2.887	2.857
17–18	3.378	3.654	3.491	2.774	2.308	1.636	1.296	1.303	1.683	2.186	2.721	2.503
18–19	1.999	3.187	3.476	3.031	2.520	1.941	1.752	1.692	2.084	2.350	1.233	1.053
19–20	581	897	1.670	2.801	2.431	2.049	2.032	2.098	2.276	1.470	421	401
20–21	0	0	0	1.739	1.930	2.027	2.316	2.285	1.279	0	0	0
21–22	516	504	427	151	1.013	1.998	2.466	1.680	783	526	743	565
22–23	2.652	2.794	2.333	1.784	2.051	2.676	3.062	2.737	3.163	2.789	3.026	2.515
23–24	6.059	6.107	5.130	3.725	3.986	4.472	4.853	4.435	5.069	4.683	5.631	5.156

**Table 11:** Electrical availability forecast (MW) from 0 to 24 h in 2022.

**Hour**	**Jan.**	**Feb.**	**Mar.**	**Apr.**	**May.**	**Jun.**	**Jul.**	**Aug.**	**Sep.**	**Oct.**	**Nov.**	**Dec.**
00–01	8.455	8.163	6.911	5.576	5.263	5.558	6.031	6.038	6.115	5.853	7.419	7.008
01–02	10.231	9.909	8.437	6.992	6.413	6.744	7.413	7.512	7.196	6.871	8.735	8.566
02–03	11.404	11.010	9.386	7.894	7.268	7.723	8.569	8.653	8.071	7.635	9.598	9.619
03–04	11.875	11.377	9.738	8.277	7.668	8.210	9.207	9.297	8.493	8.003	9.966	10.089
04–05	11.891	11.376	9.773	8.381	7.827	8.444	9.574	9.653	8.754	8.167	10.021	10.132
05–06	11.263	10.758	9.179	8.009	7.552	8.275	9.490	9.584	8.591	7.919	9.520	9.617
06–07	9.222	8.625	7.277	6.367	6.292	7.326	8.459	8.586	7.318	6.560	7.660	7.880
07–08	5.623	4.848	4.531	4.129	4.564	5.668	7.114	7.412	5.263	4.116	4.663	4.859
08–09	3.573	2.969	2.455	2.505	2.772	4.096	5.546	6.052	4.381	2.995	3.000	3.147
09–10	1.927	1.365	807	884	1.413	2.486	3.452	3.985	2.904	1.827	1.518	1.498
10–11	1.045	806	330	229	844	1.576	2.218	2.665	2.094	1.182	899	608
11–12	910	832	276	56	501	1.003	1.478	1.688	1.372	678	810	537
12–13	1.210	1.065	536	16	180	395	523	751	589	246	819	810
13–14	1.199	1.050	679	0	0	0	0	33	0	0	668	776
14–15	1.807	1.729	1.395	630	541	319	36	0	332	490	1.267	1.247
15–16	2.743	2.668	2.329	1.676	1.474	1.068	640	601	1.051	1.347	2.138	2.141
16–17	3.396	3.284	3.038	2.365	2.056	1.433	1.018	994	1.388	1.852	2.742	2.714
17–18	3.322	3.567	3.400	2.806	2.316	1.624	1.257	1.289	1.665	2.123	2.638	2.418
18–19	2.028	3.155	3.407	3.064	2.524	1.932	1.723	1.693	2.084	2.331	1.263	1.086
19–20	624	913	1.628	2.817	2.409	2.012	1.983	2.105	2.286	1.496	461	435
20–21	0	0	0	1.769	1.895	1.967	2.248	2.306	1.335	76	0	0
21–22	529	511	452	274	1.042	1.967	2.420	1.780	883	595	737	580
22–23	2.693	2.827	2.347	1.913	2.138	2.733	3.099	2.869	3.256	2.825	3.030	2.550
23–24	6.231	6.247	5.203	3.810	4.027	4.504	4.871	4.525	5.110	4.668	5.659	5.231

**Table 12:** Electrical availability forecast (MW) from 0 to 24 h in 2023.

**Hour**	**Jan.**	**Feb.**	**Mar.**	**Apr.**	**May.**	**Jun.**	**Jul.**	**Aug.**	**Sep.**	**Oct.**	**Nov.**	**Dec.**
00–01	8.556	8.230	6.933	5.650	5.231	5.499	6.008	6.107	6.086	5.835	7.372	7.005
01–02	10.258	9.908	8.398	7.016	6.322	6.625	7.331	7.538	7.111	6.796	8.618	8.471
02–03	11.379	10.959	9.300	7.879	7.147	7.577	8.457	8.656	7.964	7.536	9.438	9.464
03–04	11.807	11.283	9.616	8.236	7.531	8.053	9.085	9.289	8.375	7.895	9.783	9.895
04–05	11.786	11.249	9.627	8.321	7.683	8.283	9.452	9.641	8.638	8.058	9.821	9.904
05–06	11.118	10.598	9.002	7.927	7.398	8.108	9.364	9.567	8.474	7.806	9.303	9.362
06–07	9.038	8.433	7.074	6.270	6.136	7.159	8.340	8.569	7.217	6.466	7.428	7.600
07–08	5.403	4.630	4.304	4.043	4.402	5.497	6.998	7.392	5.201	4.073	4.441	4.573
08–09	3.444	2.823	2.253	2.433	2.655	4.002	5.496	6.059	4.373	3.031	2.822	2.945
09–10	1.780	1.216	613	816	1.331	2.423	3.428	4.005	2.902	1.838	1.336	1.299
10–11	945	713	203	215	833	1.577	2.260	2.730	2.145	1.229	764	456
11–12	830	757	169	66	516	1.024	1.535	1.764	1.432	720	691	405
12–13	1.129	981	441	34	197	414	564	815	631	276	694	671
13–14	1.095	938	574	0	0	0	29	81	0	0	512	619
14–15	1.655	1.561	1.243	586	493	262	0	0	270	432	1.064	1.045
15–16	2.612	2.518	2.190	1.653	1.436	1.013	593	582	986	1.296	1.953	1.953
16–17	3.306	3.163	2.924	2.374	2.047	1.399	990	979	1.342	1.823	2.597	2.571
17–18	3.267	3.479	3.308	2.838	2.325	1.613	1.248	1.290	1.648	2.126	2.555	2.333
18–19	2.056	3.122	3.337	3.097	2.528	1.924	1.723	1.710	2.085	2.378	1.293	1.119
19–20	667	928	1.587	2.834	2.388	1.975	1.964	2.126	2.295	1.588	501	470
20–21	0	0	0	1.800	1.860	1.907	2.210	2.342	1.391	218	0	0
21–22	542	519	476	399	1.072	1.935	2.404	1.894	983	730	731	595
22–23	2.735	2.860	2.362	2.043	2.224	2.791	3.166	3.016	3.349	2.926	3.035	2.585
23–24	6.403	6.387	5.275	3.896	4.067	4.535	4.918	4.629	5.151	4.718	5.686	5.306

**Table 13:** EV amount forecast that can be recharged simultaneously from 0 to 24 h in 2020.

**Hour**	**Jan.**	**Feb.**	**Mar.**	**Apr.**	**May.**	**Jun.**	**Jul.**	**Aug.**	**Sep.**	**Oct.**	**Nov.**	**Dec.**
00–01	165.034	160.547	137.315	108.942	106.505	113.555	122.717	119.245	123.459	121.901	150.253	140.262
01–02	203.538	198.238	170.269	139.230	131.881	139.665	152.700	150.448	147.302	144.563	179.389	175.108
02–03	229.081	222.227	191.164	158.854	150.235	160.295	177.005	174.198	165.695	160.785	198.330	198.555
03–04	240.190	231.307	199.654	167.549	158.830	170.501	190.193	187.514	174.549	168.509	206.664	209.580
04–05	242.030	232.601	201.313	170.408	162.276	175.318	197.549	194.819	179.702	171.886	208.443	211.748
05–06	231.096	221.543	190.667	163.854	157.191	172.188	196.025	193.610	176.472	167.032	199.102	202.585
06–07	191.849	180.181	153.645	131.580	132.089	153.203	175.131	173.663	150.411	139.131	162.473	168.773
07–08	121.273	105.653	99.704	86.412	97.778	120.234	148.062	150.314	107.729	88.167	102.151	108.633
08–09	76.632	65.205	57.188	53.357	60.108	85.687	114.091	122.029	87.911	62.618	67.093	71.066
09–10	44.404	33.271	23.892	20.740	31.563	52.264	71.146	80.154	58.127	40.231	37.633	37.926
10–11	24.908	19.815	11.665	5.529	17.329	31.457	43.857	51.918	39.832	25.939	23.371	18.203
11–12	21.426	19.648	9.785	1.101	9.412	19.231	28.462	31.961	25.053	16.010	20.978	16.020
12–13	27.446	24.640	14.556	0	2.902	7.178	10.006	13.701	10.094	7.887	21.390	21.729
13–14	28.155	25.481	17.765	373	0	0	0	0	0	4.149	19.583	21.800
14–15	42.241	41.333	33.993	14.691	12.767	8.665	3.361	1.256	9.102	16.227	33.465	33.019
15–16	60.093	59.390	52.175	34.828	30.970	23.578	15.829	14.045	23.616	33.121	50.160	50.314
16–17	71.503	70.497	65.307	47.300	41.439	30.023	22.642	21.703	29.641	42.311	60.640	60.004
17–18	68.668	74.843	71.661	55.191	45.994	32.946	26.689	26.998	34.004	46.492	56.065	51.759
18–19	39.403	64.401	70.924	60.305	50.307	38.998	35.623	34.458	41.658	48.895	24.046	20.397
19–20	10.765	17.625	34.229	56.021	49.065	41.707	41.618	42.502	45.345	30.417	7.609	7.331
20–21	0	0	0	34.513	39.303	41.733	47.675	45.935	24.460	0	0	0
21–22	10.065	9.921	8.060	865	19.671	40.610	50.220	32.267	13.669	10.657	14.969	11.006
22–23	52.219	55.235	46.364	33.427	39.282	52.361	60.510	52.754	61.410	56.612	60.424	49.609
23–24	117.739	119.327	101.152	73.132	78.923	88.813	96.706	87.580	100.576	95.511	112.076	101.612

**Table 14:** EV amount forecast that can be recharged simultaneously from 0 to 24 h in 2021.

**Hour**	**Jan.**	**Feb.**	**Mar.**	**Apr.**	**May.**	**Jun.**	**Jul.**	**Aug.**	**Sep.**	**Oct.**	**Nov.**	**Dec.**
00–01	167.065	161.900	137.764	110.065	105.880	112.362	121.669	119.670	122.878	118.712	149.318	140.209
01–02	204.079	198.211	169.502	139.368	130.066	137.274	150.477	150.012	145.611	140.223	177.043	173.213
02–03	228.579	221.211	189.441	158.204	147.801	157.375	174.191	173.301	163.559	155.975	195.144	195.467
03–04	238.843	229.424	197.211	166.377	156.092	167.354	187.164	186.394	172.201	163.517	202.994	205.684
04–05	239.929	230.058	198.389	168.853	159.405	172.095	194.513	193.609	177.389	166.853	204.434	207.189
05–06	228.183	218.350	187.126	161.856	154.116	168.848	192.916	192.313	174.143	161.939	194.753	197.467
06–07	188.149	176.338	149.592	129.296	128.967	149.861	172.158	172.364	148.389	134.403	157.835	163.183
07–08	116.868	101.304	95.162	84.333	94.532	116.801	145.168	148.946	106.494	84.476	97.708	102.911
08–09	74.048	62.292	53.144	51.567	57.773	83.807	112.506	121.207	87.762	60.496	63.544	67.008
09–10	41.471	30.287	20.015	19.042	29.915	50.995	70.090	79.592	58.100	37.620	33.994	33.946
10–11	22.903	17.965	9.133	4.894	17.107	31.485	44.110	52.274	40.857	24.029	20.676	15.178
11–12	19.815	18.143	7.650	949	9.717	19.647	29.013	32.526	26.250	14.017	18.589	13.378
12–13	25.821	22.969	12.643	0	3.246	7.543	10.237	14.026	10.936	5.639	18.889	18.960
13–14	26.067	23.241	15.673	22	0	0	0	0	0	1.310	16.471	18.662
14–15	39.195	37.960	30.950	13.477	11.798	7.525	2.045	297	7.870	12.244	29.405	28.979
15–16	57.476	56.377	49.382	34.011	30.221	22.469	14.312	12.702	22.320	29.268	46.461	46.563
16–17	69.710	68.083	63.032	47.136	41.276	29.343	21.498	20.457	28.705	38.906	57.740	57.142
17–18	67.557	73.089	69.826	55.487	46.160	32.716	25.917	26.056	33.655	43.711	54.413	50.060
18–19	39.978	63.746	69.530	60.624	50.393	38.824	35.038	33.832	41.671	46.993	24.650	21.059
19–20	11.620	17.938	33.398	56.015	48.627	40.970	40.644	41.965	45.529	29.407	8.412	8.020
20–21	0	0	0	34.782	38.605	40.537	46.319	45.693	25.580	0	0	0
21–22	10.327	10.075	8.546	3.012	20.258	39.970	49.311	33.600	15.667	10.515	14.852	11.306
22–23	53.044	55.888	46.656	35.677	41.017	53.513	61.249	54.735	63.264	55.790	60.514	50.305
23–24	121.181	122.133	102.602	74.503	79.727	89.444	97.061	88.709	101.389	93.670	112.625	103.116

**Table 15:** EV amount forecast that can be recharged simultaneously from 0 to 24 h in 2022.

**Hour**	**Jan.**	**Feb.**	**Mar.**	**Apr.**	**May.**	**Jun.**	**Jul.**	**Aug.**	**Sep.**	**Oct.**	**Nov.**	**Dec.**
00–01	169.096	163.252	138.213	111.517	105.255	111.169	120.621	120.757	122.297	117.052	148.383	140.155
01–02	204.620	198.185	168.736	139.834	128.252	134.884	148.253	150.238	143.920	137.413	174.697	171.319
02–03	228.076	220.194	187.717	157.883	145.368	154.455	171.376	173.065	161.422	152.694	191.957	192.378
03–04	237.496	227.542	194.768	165.534	153.355	164.208	184.134	185.937	169.853	160.054	199.324	201.788
04–05	237.827	227.515	195.466	167.627	156.534	168.873	191.477	193.062	175.076	163.350	200.424	202.630
05–06	225.270	215.156	183.586	160.187	151.040	165.507	189.806	191.677	171.813	158.375	190.405	192.350
06–07	184.450	172.496	145.540	127.341	125.845	146.518	169.185	171.728	146.367	131.204	153.198	157.592
07–08	112.463	96.956	90.621	82.584	91.286	113.369	142.275	148.240	105.259	82.314	93.266	97.189
08–09	71.464	59.379	49.100	50.106	55.438	81.927	110.922	121.048	87.612	59.903	59.995	62.949
09–10	38.538	27.303	16.138	17.673	28.268	49.726	69.034	79.693	58.073	36.538	30.355	29.965
10–11	20.898	16.115	6.600	4.588	16.885	31.513	44.362	53.293	41.882	23.649	17.981	12.154
11–12	18.204	16.638	5.514	1.126	10.021	20.063	29.564	33.754	27.447	13.554	16.200	10.735
12–13	24.197	21.299	10.729	329	3.590	7.908	10.468	15.014	11.779	4.921	16.388	16.191
13–14	23.980	21.002	13.580	0	0	0	0	662	0	0	13.359	15.523
14–15	36.149	34.586	27.907	12.591	10.829	6.384	728	0	6.637	9.791	25.346	24.940
15–16	54.860	53.364	46.589	33.522	29.472	21.359	12.795	12.022	21.024	26.943	42.762	42.811
16–17	67.916	65.670	60.757	47.301	41.112	28.663	20.355	19.872	27.770	37.031	54.840	54.279
17–18	66.446	71.334	67.991	56.111	46.326	32.486	25.145	25.777	33.306	42.459	52.761	48.362
18–19	40.554	63.090	68.135	61.272	50.479	38.650	34.454	33.869	41.685	46.620	25.254	21.722
19–20	12.476	18.251	32.567	56.339	48.188	40.234	39.670	42.091	45.713	29.926	9.215	8.710
20–21	0	0	0	35.380	37.907	39.341	44.963	46.113	26.701	1.529	0	0
21–22	10.588	10.230	9.031	5.488	20.845	39.330	48.402	35.595	17.665	11.904	14.734	11.605
22–23	53.869	56.541	46.947	38.256	42.752	54.666	61.989	57.378	65.118	56.498	60.605	51.001
23–24	124.623	124.939	104.053	76.203	80.530	90.075	97.415	90.500	102.202	93.358	113.173	104.620

**Table 16:** EV amount forecast that can be recharged simultaneously from 0 to 24 h in 2023.

**Hour**	**Jan.**	**Feb.**	**Mar.**	**Apr.**	**May.**	**Jun.**	**Jul.**	**Aug.**	**Sep.**	**Oct.**	**Nov.**	**Dec.**
00–01	171.127	164.605	138.662	112.991	104.630	109.975	120.162	122.140	121.715	116.702	147.448	140.102
01–02	205.161	198.158	167.970	140.322	126.437	132.493	146.619	150.760	142.230	135.912	172.350	169.424
02–03	227.574	219.178	185.993	157.584	142.934	151.535	169.150	173.127	159.286	150.723	188.770	189.290
03–04	236.148	225.660	192.325	164.713	150.618	161.062	181.693	185.776	167.506	157.901	195.654	197.893
04–05	235.726	224.972	192.542	166.423	153.663	165.651	189.030	192.811	172.764	161.156	196.414	198.071
05–06	222.356	211.963	180.045	158.541	147.965	162.167	187.285	191.339	169.483	156.121	186.056	187.232
06–07	180.750	168.653	141.487	125.408	122.723	143.175	166.800	171.389	144.346	129.315	148.560	152.002
07–08	108.059	92.607	86.079	80.856	88.041	109.936	139.970	147.831	104.024	81.462	88.823	91.467
08–09	68.881	56.466	45.056	48.668	53.104	80.048	109.925	121.185	87.462	60.621	56.446	58.890
09–10	35.605	24.318	12.261	16.327	26.621	48.457	68.566	80.091	58.046	36.766	26.716	25.985
10–11	18.893	14.266	4.068	4.304	16.663	31.542	45.203	54.608	42.907	24.579	15.286	9.129
11–12	16.593	15.133	3.378	1.325	10.325	20.479	30.703	35.278	28.644	14.401	13.811	8.093
12–13	22.572	19.628	8.815	680	3.935	8.273	11.288	16.298	12.621	5.512	13.887	13.422
13–14	21.892	18.763	11.488	0	0	0	588	1.621	0	0	10.248	12.385
14–15	33.103	31.213	24.864	11.727	9.861	5.244	0	0	5.405	8.647	21.286	20.900
15–16	52.243	50.351	43.797	33.056	28.723	20.250	11.867	11.637	19.727	25.929	39.063	39.059
16–17	66.123	63.257	58.483	47.488	40.949	27.984	19.799	19.585	26.834	36.465	51.940	51.416
17–18	65.335	69.579	66.156	56.758	46.492	32.256	24.962	25.794	32.957	42.517	51.109	46.663
18–19	41.129	62.435	66.740	61.943	50.565	38.476	34.457	34.203	41.698	47.556	25.859	22.385
19–20	13.332	18.565	31.737	56.684	47.750	39.497	39.284	42.513	45.898	31.755	10.018	9.399
20–21	0	0	0	36.000	37.208	38.145	44.196	46.830	27.821	4.369	0	0
21–22	10.850	10.385	9.516	7.986	21.432	38.690	48.082	37.887	19.662	14.602	14.617	11.904
22–23	54.694	57.195	47.239	40.856	44.487	55.819	63.317	60.318	66.971	58.515	60.696	51.697
23–24	128.066	127.745	105.503	77.926	81.334	90.706	98.358	92.588	103.014	94.355	113.722	106.124

## 2 Experimental design, materials, and methods

Is applied linear forecasting technique between the years 2007 to 2019 in the file Resume_Raw_Data.xlsx, The mathematical equation for developing the linear forecast is  *a*+bx, $a=\bar{y}-b\bar{x}$, $b=\frac{\sum \left(x-\bar{x\;}\right)\left(y-\bar{y\;}\right)}{\sum {\left(x-\bar{x}\right)}^{2}}$ where x and y are the sample means Average(electric-demand) and Average(year). In the file Resume_Raw_Data.xlsx is visualized the process to obtain the linear forecast.

On the other hand, the maximum value per month, located in every column of the Table 5, Table 6, Table 7, Table 8 minus the demand value recorded within each time band per day represents electricity availability in Megawatts for the years from 2020 to 2023 shown each of them in Table 9, Table 10, Table 11, Table 12.

**Fig. 1:**
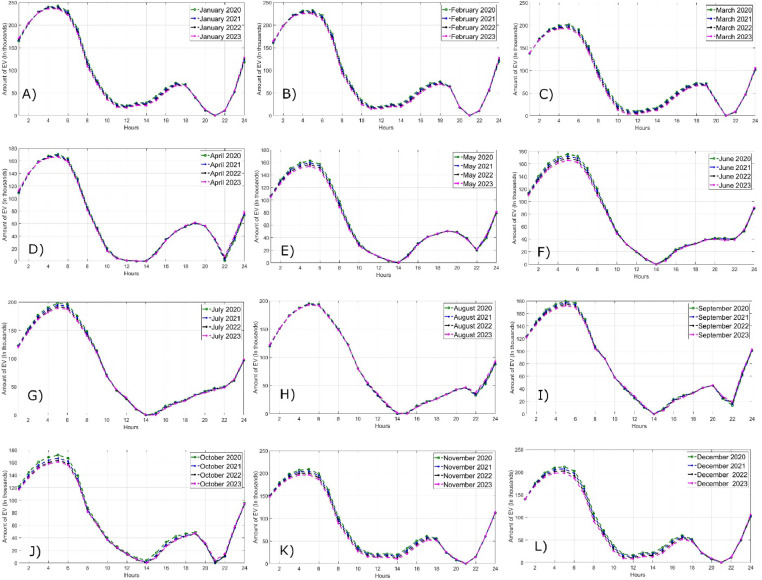
EV amount forecast that can be recharged simultaneously from 0 to 24 h in 2020,2021,2022,2023 per month A) January B) February C) March D) April E) May F) June G) July H) August I) September J) October K) November L) December.

The values in the Table 13,14,15,16 were obtained by dividing every data of the Tables 9,10,11,12 per 50 kW. Each result determines the number of new EVs that could be recharged using a fast charge in 60-min. intervals with a connection power of 50 kW for the forecasted years 2020, 2021, 2022, 2023 respectively. The summary of Tables 13,14,15,16 shows the forecast for 2020–2023 in graphs per month for easy reading in the Fig. 1.

The first two months of the year 2020 recorded values close to the linear forecast, with an error of less than 4% in January (a maximum of 3.8% in the 23 to 24 h), and a maximum error of 4.5% in February (in the 18 to 19 h interval). However, in March 2020, the error rose to 10% due to the decrease in national electricity demand in Spain as a result of the COVID-19, especially in the 7–11 a.m. time slot. The maximum error peaks are highlighted with green color in Table 17.

**Table 17:** Margin of error between forecast and actual value of the consumption demand of the Spanish electricity grid.

		**January**			**February**			**March**	
**Hour**	**Real**	**Forecast**	**%Error**	**Real**	**Forecast**	**%Error**	**Real**	**Forecast**	**%Error**
00–01	27.293	26.383	3,34	27.293	26.850	1,62	24.721	25.500	−3,15
01–02	25.180	24.458	2,87	25.180	24.965	0,85	23.161	23.852	−2,98
02–03	23.798	23.180	2,60	23.798	23.766	0,14	22.098	22.808	−3,21
03–04	23.109	22.625	2,10	23.109	23.312	−0,88	21.530	22.383	−3,96
04–05	22.877	22.533	1,50	22.877	23.247	−1,62	21.387	22.300	−4,27
05–06	23.274	23.080	0,83	23.274	23.800	−2,26	21.675	22.832	−5,34
06–07	25.137	25.042	0,38	25.137	25.868	−2,91	23.243	24.684	−6,20
07–08	28.519	28.571	−0,18	28.519	29.595	−3,77	25.101	27.381	−9,08
08–09	31.044	30.803	0,78	31.044	31.617	−1,85	26.664	29.506	−10,66
09–10	32.597	32.414	0,56	32.597	33.214	−1,89	28.316	31.171	−10,08
10–11	33.865	33.389	1,41	33.865	33.886	−0,06	29.410	31.783	−8,07
11–12	34.166	33.563	1,76	34.166	33.895	0,79	29.941	31.877	−6,46
12–13	33.883	33.262	1,83	33.883	33.645	0,70	30.154	31.638	−4,92
13–14	33.857	33.227	1,86	33.857	33.603	0,75	30.466	31.478	−3,32
14–15	33.093	32.522	1,72	33.093	32.810	0,85	29.856	30.666	−2,71
15–16	32.173	31.630	1,69	32.173	31.908	0,82	28.417	29.757	−4,72
16–17	31.654	31.059	1,88	31.654	31.352	0,95	27.465	29.100	−5,96
17–18	31.671	31.201	1,48	31.671	31.135	1,69	27.057	28.783	−6,38
18–19	33.149	32.664	1,46	33.149	31.657	4,50	27.178	28.820	−6,04
19–20	34.580	34.096	1,40	34.580	33.996	1,69	28.923	30.654	−5,99
20–21	35.201	34.634	1,61	35.201	34.877	0,92	30.894	32.366	−4,76
21–22	34.866	34.131	2,11	34.866	34.381	1,39	30.766	31.963	−3,89
22–23	32.698	32.024	2,06	32.698	32.115	1,78	28.717	30.048	−4,63
23–24	29.884	28.748	3,80	29.884	28.911	3,26	26.405	27.308	−3,42

## Declaration of Competing Interest

The authors declare that they have no known competing financial interests or personal relationships which have, or could be perceived to have, influenced the work reported in this article.

## Appendix Supplementary materials

Image, application 1
